# Sociodemographic patterns in pharmacy dispensing of medications for erectile dysfunction in Sweden

**DOI:** 10.1007/s00228-017-2361-9

**Published:** 2017-11-03

**Authors:** Pernilla J. Bjerkeli, Shai Mulinari, Sofia Zettermark, Juan Merlo

**Affiliations:** 1 Unit for Social Epidemiology, Faculty of Medicine, CRC, Lund University, Jan Waldenströms gata 35, 205 02 Malmö, Sweden; 2 Department for Biomedicine and Public Health Research, University of Skövde, Box 408, SE 541 28 Skövde, Sweden; 3 Department of Sociology, Faculty of Social Sciences, Lund University, Box 114, SE-221 00 Lund, Sweden

**Keywords:** Erectile dysfunction, Pharmacoepidemiology, Drug utilisation research, Masculinity, Phosphodiesterase type 5 inhibitors

## Abstract

**Purpose:**

The purpose of this study is to investigate the relationship between sociodemographic factors and pharmacy dispensing of medications for erectile dysfunction (ED) in the general population of middle-aged and elderly men. By considering a number of medical conditions that could promote or contraindicate use of ED medication, the analysis could help capture prescription patterns that might not be explained by medical needs.

**Methods:**

Individual-level pharmacy dispensing data from 2006 for a population-based cohort of 216,148 men aged 45–79 years in the county Scania, Sweden, were analysed. Multiple logistic regression was applied, and area under the receiver operating characteristic curve (AUC) was calculated to quantify the discriminatory accuracy (DA) of the associations. National trends in pharmacy dispensing of ED medication between 2006 and 2016 were also analysed.

**Results:**

Pharmacy dispensing of ED medication increased between 2006 and 2016, particularly among men aged 65–79 years (from 6.8 to 9.2%). Dispensing of ED medication was positively associated with higher socioeconomic position, and divorced and widowed men were more likely to fill a prescription with ED medication than married men. These associations remained after adjusting for medical conditions. The DA of the associations was, however, rather low (AUC = 0.69 among 45–64 year olds and AUC = 0.65 among 65–79 year olds).

**Conclusions:**

Pharmacy dispensing of ED medication seem linked to the individuals socioeconomic position, age and marital status suggesting sociodemographic disparities in the pharmacy dispensing targeting sexual function. However, the low DA of the associations shows the limited capacity of these factors to predict ED medication use at the individual level.

## Introduction

Erectile dysfunction (ED) is usually defined as the persistent inability to attain or maintain an erection sufficient for satisfactory sexual performance [1]. The prevalence increases with increasing age, ranging from 2 to 9% in men under the age of 40 years and up to 86% in men 80 years and older [2–4]. For men who wish to treat their ED, there are pharmaceutical preparations available, the most common ones being the phosphodiesterase type 5 inhibitors (PDE5I) [5], for example Sildenafil.

The use of ED medication differs in different parts of the world [6]. A multinational study including eight different countries indicated that the odds of receiving treatment for ED differed between countries, being highest in the UK, lower in the USA and lowest in France [6]. Swedish data show that approximately 5% of Swedish men filled a prescription of an ED medication in 2006 [7]. The prevalence of ED medication use increases with increasing age up till around the age of 50–60 years, after that, it decreases [6, 8]. It is also more common among men who report having a regular sexual partner than among those without sexual partner [8–10].

Several studies have shown that the use of ED medication is more common among men with higher socioeconomic position than among those with lower socioeconomic position in Sweden [7, 9–11] and elsewhere [8, 12]. This socioeconomic gradient is interesting considering that the prevalence of ED goes in the opposite direction, being higher among men with low socioeconomic position [13]. As patients have to pay for most ED medications, including the PDE5I, out of their own pocket in Sweden, affordability may contribute to socioeconomic differences.

Direct-to-consumer advertising of prescription drugs is banned in Europe, but research has shown that companies have exploited the legality of so-called disease awareness campaigns to market ED medications to the public in Sweden [14]. Analyses of disease awareness campaigns also show how the promotion relates the use of ED medications to culturally grounded ideals of masculinity, ageing and high socioeconomic status [15, 16]. However, little is known about how these ideals are reflected in the actual pharmacy dispensing patterns of ED medication. In fact, rather little is known about the social pharmacoepidemiology of these medications. Studies are available from Sweden [7, 9–11] and elsewhere [5, 6, 8, 17–20]. Many of them, however, are based on selected populations from commercial insurance plans [5, 19], or specific disease groups [9, 10]. Others have analysed use of ED medications without considering disease-related factors or use of other medications [7, 11].

In this study, population-based pharmacy dispensing data on the individual and aggregated level were combined to analyse pharmacy dispensing patterns of ED medication in Sweden. The aim was to investigate the relationship between sociodemographic factors and pharmacy dispensing of ED medications in the general population of middle-aged (45–64 years) and elderly (65–79 years) men. When doing so, a number of known medical conditions that could promote or contraindicate use of ED medication were considered. Examples of such factors are hypertension, obesity, diabetes mellitus, dyslipidaemia and depression [13, 21–24]. In this way, the analysis could help to capture pharmacy dispensing patterns that might not be explained by medical needs.

## Methods

### Data sources and study population

Both national- and regional-level analyses were performed. Information on pharmacy dispensing was in both cases from the Swedish Prescribed Drug Register (SPDR) [25], which is managed by the Swedish National Board of Health and Welfare (NBHW). The SPDR records information on all prescription fills at Swedish pharmacies from July 2005 and onwards.

The national-level analyses were based on an online publically available database which is administered by NBHW and contains aggregated-level information on pharmacy dispensing according to The Anatomical Therapeutic Chemical (ATC) Classification System [http://www.socialstyrelsen.se/statistik/statistikdatabas/lakemedel]. This database was used to investigate age-stratified, national trends in pharmacy dispensing of ED medication 2006–2016.

The regional-level analyses were based on individual-level information from the Longitudinal Multilevel Analysis (LOMAS) database. LOMAS is a research database approved by the Regional Ethical Review Board in Lund, Sweden, and by the data safety committees at Statistics Sweden and at the NBHW. The database contains record linkage information about pharmacy dispensing from the SPDR, sociodemographic factors from the longitudinal integration database for health insurance and labour market studies (LISA) and hospital discharge diagnoses from the National Patient Register (NPR). From the LOMAS database, all 216,148 men aged 45–79 years who were residents in the county Scania, in south Sweden, by December 31, 2005, were selected. The age group was chosen in order to cover ages with the highest prevalence of ED medication use.

### Assessment of variables

#### Outcome variables

In the national-level analyses, the outcome variable was aggregated information on pharmacy dispensing of all ED medications defined by the ATC code G04BE (Alprostadil, Sildenafil, Tadalafil, Vardenafil, Avanafil and combinations) and expressed as the number of users per thousand inhabitants per year between 2006 and 2016.

In the individual-level analyses, the outcome variable was pharmacy dispensing of ED medication (yes or no), defined as at least one prescription fill of a PDE5I (ATC code G04BE03 Sildenalfil, G04BE08 Tadalafil, G04BE09 Vardenafil and G04BE10 Avanafil) during 2006.

#### Sociodemographic variables

The sociodemographic variables were defined in 2004.

A composite measure of socioeconomic position was created by including both information on the highest achieved educational level (compulsory school 9 years or less, secondary education 3 years or less and higher education) and disposable individual income in tertiles. The nine categories of the combined measure were (i) low income low education, (ii) low income medium education, (iii) low income high education, (iv) medium income low education, (v) medium income medium education, (vi) medium income high education, (vii) high income low education, (viii) high income medium education and (ix) high income high education. The first category (i.e. low income low education) was used as reference category.

Four categories of marital status (married, unmarried, divorced and widowed) were defined and the married category was used as reference.

As previously described elsewhere [26], country of birth was categorised according to the World Bank classification of the world’s economies, which is based on gross national income, into the following categories: Sweden, other high-income economies, upper middle-income economies and lower middle- and low-income economies. Sweden was used as reference category.

#### Diagnoses and other medication

Our study aimed to identify use of ED medication that was conditioned by sociodemographic factors rather than by medical conditions that might indicate or contraindicate the use of ED medication. Therefore, for the statistical analyses (see under), information on prescription fills of testosterone (ATC code G03BA03), antihypertensive medication (ATC codes C02, C03, C07, C08 and C09), insulin (ATC code A10A), oral antidiabetics (ATC code A10B), vasodilators (ATC code C01D), hypnotic or sedative medication (ATC code N05C), antidepressant medication (ATC code N06A) and anti-obesity medication (ATC code A08A) during 2006 was obtained. Pharmacy dispensing was defined as having filled a prescription of the medication in question at a pharmacy during the study period. Data on hospital discharges with a diagnosis of ischemic heart disease (ICD10 codes I20–I25), diabetes (ICD10 codes E10–E14), prostate cancer (ICD10 code C61) or prostatic hyperplasia (ICD10 code N40) between 2001 and 2005 were included (primary and two secondary diagnoses).

### Statistical analysis

The study population was stratified into two age groups (45–64 and 65–79 years) according to the normal retirement age in Sweden (65 years). Age-stratified logistic regression analyses were performed to obtain odds ratios (OR) and 95% confidence intervals (CI) of the association between sociodemographic factors and the use of ED medication.

In order to identify the use of ED medication that was conditioned by sociodemographic factors over and above medical conditions that might indicate or contraindicate the use of ED medication, the analyses were adjusted by the information on diagnoses and other medication described above. For doing so in a parsimonious way rather than entering all the variables in a model, a risk score variable was created. This risk score was obtained from a separate multiple logistic regression with pharmacy dispensing of ED medication as dependent variable and the variables representing risk factors or contraindications (i.e. pharmacy dispensing of testosterone, antihypertensive medication, insulin, oral antidiabetics, vasodilators, lipid-modifying medication, sedatives and antidepressants and diagnosis of ischemic heart disease, diabetes, prostate cancer or prostate hyperplasia) as independent variables. The predicted probability of filling an ED medication prescription was calculated from the regression model and categorised into three groups by tertiles.

Four consecutive regression models were then applied with pharmacy dispensing of ED medication as dependent variable. The first model (model 1) contained only age. The second model (model 2) contained age and the risk score variable. The third model (model 3) contained age and sociodemographic variables. A fourth model (model 4) included age, the risk score variable and the sociodemographic variables. Bivariate logistic regression analysis using only one specific variable at time was performed in order to investigate the associations between each independent variable and pharmacy dispensing of ED medication.

The discriminatory accuracy (DA) of models 1–4 was analysed using the area under the receiver operating characteristic curve (AUC). This provided a measure of the regression models’ ability to discriminate between those filling and not filling a prescription for ED medication. The AUC takes a value between 1 and 0.5 where 1 is perfect discrimination and 0.5 would be as informative as flipping a coin [27] (i.e. the covariates have no predictive power). All analyses were performed with SPSS 22.0 (IBM).

## Results

In the whole country of Sweden, the proportion of 45–64-year-old men having a filled a prescription with an ED medication was rather similar in 2006 (i.e. 4.5%) as compared with 2016 (i.e. 5.3%), but among 65–79-year-old men, this figure increased from 6.7% in 2006 to 9.2% in 2016, Fig. 1.

**Fig. 1:**
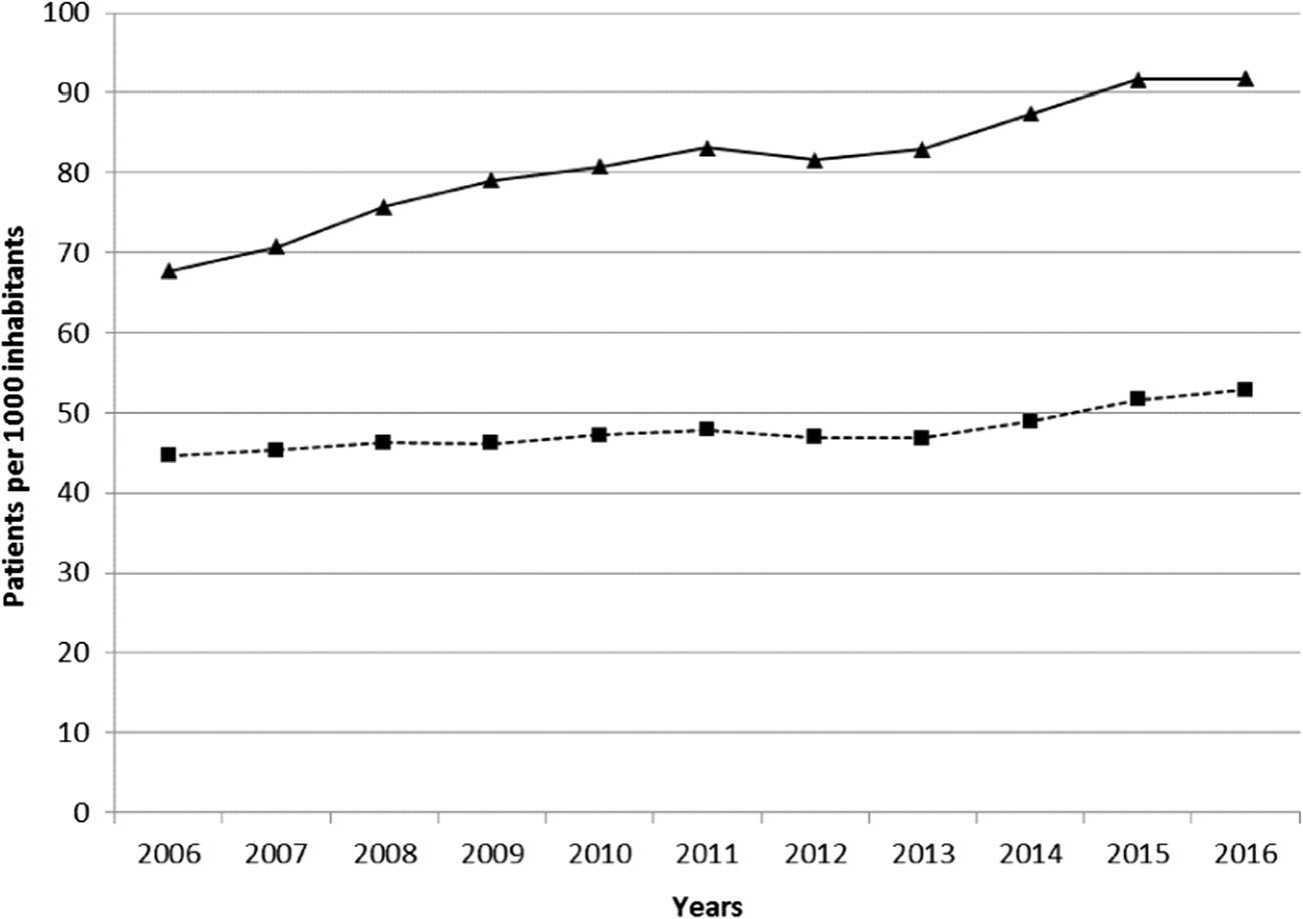
Pharmacy dispensing of medication for erectile dysfunction in men aged 45–64 years (dotted line) and 65–79 years (bold line) in Sweden. Data is from the Swedish National Board of Health and Welfares public statistics database

Data on the individual level for the county Scania in 2006 included 150,700 men aged 45–64 years and 65,448 men aged 65–79 years, Table 1. In this population, 4.7% (*n* = 7111) of the 45–64 year olds and 5.8% (*n* = 3787) of the 65–79 year olds had filled a prescription for ED medication. The highest proportion (i.e. 7.0%, *n* = 1886) was seen among those aged 65–69 years (*N* = 26,811).

**Table 1:** Prevalence of pharmacy dispensing of medication for erectile dysfunction (ED) (i.e. phosphodiesterase type 5 inhibitor (PDE5I) during 2006 among men aged 45–64 and 65–79 years residing in the county Scania, Sweden, by age, socioeconomic characteristics, medication use and hospital diagnoses. Values are numbers (*N*, *n*), percentages (%), odds ratios (OR) and 95% confidence intervals (95% CI) from bivariate logistic regressions

	45–64-year-old men						65–79-year-old men					
	Total	ED medication		OR	95% CI		Total	ED medication		OR	95% CI	
	*N*	*N*	%		Lower	Upper	*N*	*n*	%		Lower	Upper
Total	150,700	7111	4.72				65,448	3787	5.79			
Age												
45–49	37,177	925	2.49	1.00 (ref.)								
50–54	36,821	1426	3.87	1.58	1.45	1.72						
55–59	39,728	2251	5.67	2.35	2.18	2.55						
60–64	36,974	2509	6.79	2.85	2.64	3.08						
65–69							26,811	1886	7.03	1.00 (ref.)		
70–74							20,980	1230	5.86	0.82	0.76	0.89
75–79							17,657	671	3.80	0.52	0.48	0.57
Socioeconomic position												
Low income low education	11,913	493	4.14	1.00 (ref.)			14,015	530	3.78	1.00 (ref.)		
Low income medium education	16,294	685	4.20	1.02	0.90	1.14	8014	426	5.32	1.43	1.25	1.63
Low income high education	6964	285	4.09	0.99	0.85	1.15	1869	111	5.94	1.61	1.30	1.98
Medium income low education	17,782	725	4.08	0.99	0.88	1.11	5894	369	6.26	1.70	1.48	1.95
Medium income medium education	24,612	1107	4.50	1.09	0.98	1.22	6396	451	7.05	1.93	1.70	2.20
Medium income high education	8170	402	4.92	1.20	1.05	1.37	3626	292	8.05	2.23	1.92	2.58
High income low education	11,034	589	5.34	1.31	1.16	1.48	2336	197	8.43	2.34	1.98	2.78
High income medium education	24,370	1264	5.19	1.27	1.14	1.41	3929	376	9.57	2.69	2.35	3.09
High income high education	27,308	1483	5.43	1.33	1.20	1.48	4548	501	11.02	3.15	2.77	3.58
Missing on educational level	2253	78	3.46	–	–	–	14,821	534	3.60	–	–	–
Marital status												
Married	91,630	4235	4.62	1.00 (ref.)			45,012	2549	5.66	1.00 (ref.)		
Unmarried	29,192	772	2.64	0.56	0.52	0.61	5502	105	1.91	0.32	0.27	0.40
Divorced	27,877	1885	6.76	1.50	1.42	1.58	9749	690	7.08	1.27	1.16	1.38
Widowed	2001	219	10.94	2.54	2.20	2.93	5185	443	8.54	1.56	1.40	1.73
Country of birth												
Sweden	125,840	5851	4.65	1.00 (ref.)			56,755	3308	5.83	1.00 (ref.)		
Other high-income-economies	8332	413	4.96	1.07	0.97	1.19	4482	268	5.98	1.03	0.90	1.17
Upper middle-income economies	5358	294	5.49	1.19	1.06	1.34	1597	107	6.70	1.16	0.95	1.42
Low middle- and low-income economies	10,780	532	4.94	1.07	0.97	1.17	2516	101	4.01	0.68	0.55	0.83
Missing	390	21	5.38				98	3	3.06			
Pharmacy dispensing												
Testosterone	600	176	29.33	8.57	7.18	10.23	250	83	33.20	8.25	6.33	10.76
Antihypertensive medication	37,161	2875	7.74	2.16	2.06	2.27	35,555	2131	5.99	1.09	1.02	1.16
Insulin	4067	504	12.39	3.00	2.72	3.30	3413	164	4.81	0.81	0.69	0.96
Oral antidiabetics	7071	760	10.75	2.60	2.40	2.82	6340	410	6.47	1.14	1.03	1.27
Vasodilator	4284	256	5.98	1.31	1.15	1.49	7368	275	3.73	0.60	0.53	0.68
Lipid-modifying medication	20,402	1742	8.54	2.17	2.05	2.30	20,293	1256	6.19	1.11	1.04	1.19
Anti-obesity medication	1219	169	13.86	3.31	2.80	3.90	341	45	13.20	2.49	1.82	3.42
Hypnotic or sedative medication	10,550	1013	9.60	2.34	2.18	2.50	9074	621	6.84	1.24	1.13	1.35
Antidepressant	10,914	825	7.56	1.74	1.61	1.87	5701	250	4.39	0.73	0.64	0.83
Diagnosis												
Ischemic heart disease	5120	372	7.27	1.61	1.45	1.80	7648	332	4.34	0.71	0.64	0.80
Diabetes	2994	295	9.85	2.26	2.00	2.55	3924	171	4.36	0.73	0.62	0.85
Prostate cancer	336	95	28.27	8.05	6.34	10.23	414	44	10.63	1.95	1.42	2.67
Prostate hyperplasia	190	17	8.95	1.99	1.21	3.27	522	40	7.66	1.28	0.92	1.76

In both age strata, bivariate analysis showed that pharmacy dispensing of ED medication was more common among men who had a high socioeconomic position compared to those with low socioeconomic position and more common among divorced or widowed men than among married men, Table 1.

Among the middle-aged men, pharmacy dispensing of ED medications was associated with a number of variables representing risk factors for ED such as pharmacy dispensing of antihypertensive medication, insulin, oral antidiabetics, lipid-modifying medications, anti-obesity medication, hypnotics, sedatives and antidepressants and having a diagnosis of diabetes or prostate cancer at a hospital discharge, Table 1. Among the older men, however, some of the variables representing risk factors for ED were not positively associated with pharmacy dispensing of ED medication, for example pharmacy dispensing of insulin or antidepressants and having received a diabetes diagnosis. Variables representing contraindications for the use of ED medication (pharmacy dispensing of vasodilators and diagnosis of ischemic heart disease) were indeed negatively associated with pharmacy dispensing of ED medications among the older men. In the group of middle-aged men, however, these two variables were positively associated with pharmacy dispensing of ED medication. When all risk score variables were included in a multiple regression, pharmacy dispensing of vasodilators or having ischemic heart disease at hospital discharge was negatively associated with pharmacy dispensing of ED medication in both age strata, Table 2.

**Table 2:** Associations between pharmacy dispensing of medication for erectile dysfunction (ED) (i.e. phosphodiesterase type 5 inhibitors) during 2006 among men aged 45–64 years (*n* = 150,700) and 65–79 years (*n* = 65,448), residing in the county Scania, Sweden, and the variables used to create a risk score. Results from a multiple logistic regression with ED medication use as dependent variable. Values are odds ratios (OR) and 95% confidence intervals (CI)

	45–64 years			65–79 years		
	OR	95% CI		OR	95% CI	
Pharmacy dispensing						
Antihypertensive medication	1.66	1.57	1.76	1.11	1.03	1.20
Insulin	1.78	1.58	2.01	0.85	0.71	1.03
Oral antidiabetics	1.48	1.35	1.63	1.19	1.05	1.34
Vasodilator	0.65	0.56	0.75	0.57	0.50	0.65
Lipid-modifying medication	1.40	1.30	1.50	1.25	1.15	1.36
Anti-obesity medication	1.90	1.60	2.25	2.31	1.67	3.18
Hypnotics or sedatives	1.86	1.72	2.01	1.35	1.23	1.49
Antidepressant	1.20	1.11	1.31	0.66	0.58	0.76
Testosterone	6.98	5.81	8.39	7.91	6.05	10.35
Diagnosis at hospital discharge						
Ischemic heart disease	0.93	0.82	1.06	0.74	0.65	0.84
Diabetes	0.81	0.70	0.94	0.73	0.60	0.87
Prostate cancer	8.34	6.54	10.64	1.92	1.40	2.63
Prostate hyperplasia	1.87	1.13	3.11	1.26	0.91	1.74

Multiple regression showed that the risk score variable combined with age (model 2) as well as the sociodemographic variables combined with age (model 3) was associated with pharmacy dispensing of ED medication in both age groups (Tables 3 and 4). In the fourth model (model 4), the variables conclusively associated with pharmacy dispensing of ED medication in both age groups were age, the risk score variable, socioeconomic position and marital status. Country of birth was only associated with pharmacy dispensing of ED medication among the middle-aged men. The odds ratios for the sociodemographic variables and the risk score variable were rather unaltered in model 4, compared to models 2 and 3, suggesting that the risk score and the sociodemographic variables both have independent effects.

**Table 3:** Associations between on the one hand, pharmacy dispensing of medication for erectile dysfunction (ED) (i.e. phosphodiesterase type 5 inhibitors) and on the other hand, age, risk score for pharmacy dispensing of ED medication^a^ and socioeconomic characteristics in a population sample of men aged 45–64 years (*n* = 148,447) in Scania, Sweden. Results from multiple logistic regressions. Values are odds ratios (OR) and 95% confidence intervals (CI)

	Model 1			Model 2			Model 3			Model 4		
	OR	95% CI		OR	95% CI		OR	95% CI		OR	95% CI	
Age												
45–49	1.00 (ref.)			1.00 (ref.)			1.00 (ref.)			1.00 (ref.)		
50–54	1.58	1.45	1.72	1.43	1.32	1.56	1.54	1.42	1.68	1.40	1.28	1.20
55–59	2.35	2.18	2.54	1.93	1.78	2.09	2.27	2.10	2.46	1.86	1.72	2.02
60–64	2.85	2.64	3.08	2.13	2.97	2.31	2.76	2.55	2.99	2.07	1.91	2.24
Risk score^a^												
1st tertile				1.00 (ref.)						1.00 (ref.)		
2nd tertile				1.86	1.62	2.13				1.89	1.65	2.18
3rd tertile				2.60	2.47	2.73				2.61	2.48	2.75
Socioeconomic position												
Low income low education							1.00 (ref.)			1.00 (ref.)		
Low income medium education					1.03	0.92	1.16	1.04	0.93	1.18		
Low income high education							1.01	0.87	1.24	1.07	0.92	1.24
Medium income low education					1.01	0.89	1.13	1.03	0.92	1.16		
Medium income medium education					1.18	1.06	1.32	1.23	1.10	1.37		
Medium income high education					1.31	1.15	1.51	1.39	1.21	1.59		
High income low education							1.54	1.20	1.61	1.42	1.25	1.61
High income medium education					1.40	1.26	1.56	1.48	1.33	1.65		
High income high education							1.67	1.34	1.82	1.63	1.46	1.82
Marital status												
Married							1.00 (ref.)			1.00 (ref.)		
Unmarried							0.72	0.66	0.78	0.73	0.67	0.79
Divorced							1.59	1.50	1.68	1.59	1.50	1.69
Widowed							2.18	1.88	2.52	2.16	1.87	2.51
Country of birth												
Sweden							1.00 (ref.)		1.00 (ref.)			
Other high-income economies					1.09	0.98	1.21	1.11	0.99	1.23		
Upper middle-income economies					1.31	1.16	1.49	1.31	1.16	1.49		
Low middle- and low-income economies					1.35	1.22	1.49	1.32	1.20	1.46		

^a^The risk score included variables representing medical conditions that could promote or contraindicate use of ED medication such as pharmacy dispensing of testosterone, antihypertensive medication, insulin, oral antidiabetics, vasodilators, lipid-modifying medication, sedatives and antidepressants and diagnosis of ischemic heart disease, diabetes, prostate cancer or prostate hyperplasia

**Table 4:** Associations between on the one hand, pharmacy dispensing of medication for erectile dysfunction (ED) (i.e. phosphodiesterase type 5 inhibitors) and on the other hand, age, risk score for pharmacy dispensing of ED medication^a^ and socioeconomic characteristics in a population sample of men aged 65–79 years (*n* = 50,627) in Scania, Sweden. Results from multiple logistic regressions. Values are odds ratios (OR) and 95% confidence intervals (CI)

	Model 1			Model 2			Model 3			Model 4		
	OR	95% CI		OR	95% CI		OR	95% CI		OR	95% CI	
Age												
65–69	1.00 (ref.)			1.00 (ref.)			1.00 (ref.)		1.00 (ref.)			
70–74	0.82	0.76	0.89	0.81	0.75	0.87	0.90	0.83	0.97	0.88	0.82	0.95
75–79	0.52	0.48	0.57	0.51	0.47	0.56	0.72	0.62	0.85	0.71	0.60	0.83
Risk score^a^												
1st tertile				1.00 (ref.)					1.00 (ref.)			
2nd tertile				1.23	1.03	1.46				1.24	1.02	1.51
3rd tertile				1.68	1.57	1.79				1.61	1.50	1.73
Socioeconomic position												
Low income low education					1.00 (ref.)		1.00 (ref.)					
Low income medium education					1.36	1.19	1.55	1.35	1.19	1.55		
Low income high education				1.53	1.24	1.89	1.53	1.23	1.89			
Medium income low education					1.57	1.37	1.80	1.55	1.35	1.78		
Medium income medium education				1.78	1.56	2.03	1.75	1.53	1.99			
Medium income high education					2.11	1.82	2.45	2.08	1.79	2.41		
High income low education					2.14	1.80	2.54	2.10	1.76	2.49		
High income medium education				2.45	2.13	2.82	2.38	2.07	2.74			
High income high education				2.88	2.53	3.28	2.83	2.49	3.22			
Marital status												
Married							1.00 (ref.)		1.00 (ref.)			
Unmarried						0.38	0.31	0.48	0.39	0.32	0.49	
Divorced						1.27	1.16	1.39	1.29	1.17	1.42	
Widowed						1.78	1.57	2.01	1.80	1.59	2.04	
Country of birth												
Sweden						1.00 (ref.)		1.00 (ref.)				
Other high-income economies				1.05	0.90	1.21	1.06	0.91	1.22			
Upper middle-income economies				1.17	0.94	1.45	1.18	0.95	1.47			
Low middle- and low-income economies				0.92	0.74	1.14	0.94	0.76	1.17			

^a^The risk score included variables representing medical conditions that could promote or contraindicate use of ED medication such as pharmacy dispensing of testosterone, antihypertensive medication, insulin, oral antidiabetics, vasodilators, lipid-modifying medication, sedatives and antidepressants and diagnosis of ischemic heart disease, diabetes, prostate cancer or prostate hyperplasia

**Fig. 2:**
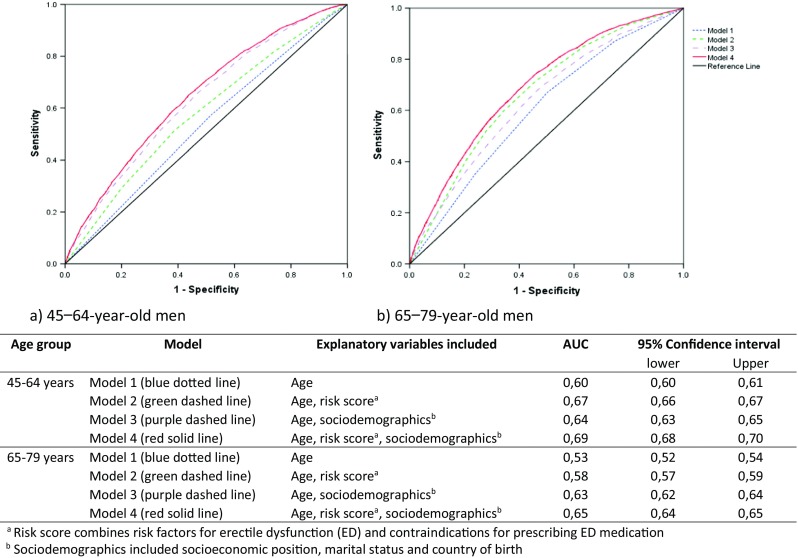
Area under the receiver operating characteristic curves for pharmacy dispensing of medication for erectile dysfunction (ED) (i.e. phosphodiesterase type 5 inhibitors) in a population sample of men aged 45–64 years (**a**) and 65–79 years (**b**) (*n* = 148,447 and *n* = 50,627, respectively). Black solid line represents AUC 0.5

The DA for model 1, including only age, was 0.60 among the middle-aged men and 0.53 among the older men, Fig. 2. For both age groups, the DA was rather low in model 2 (AUC = 0.67 among middle-aged men and AUC = 0.58 among the older men) and in model 3 (AUC = 0.64 among middle-aged men and AUC = 0.63 among the older men). Comparing model 1 and model 3 indicates that the introduction of the sociodemographic variables to the model was followed by an increase of the AUC which was greater among men above 65 years than among those aged 45–64 years. Model 4, which included both age, the risk score variable and the sociodemographic variables yielded a slightly increased DA in both age groups (AUC = 0.69 among middle-aged men and AUC = 0.65 among the older men) compared to the previous models.

## Discussion

The proportion of Swedish men filling prescriptions of ED medications increased between 2006 and 2016. In 2006, pharmacy dispensing of ED medication was positively associated with higher socioeconomic position and widowed men were more likely to fill a prescription for ED medication than married men. These associations could not entirely be explained by differences in the distribution of variables representing medical conditions that could promote or contraindicate use of ED medication, since the associations remained after controlling for these variables.

The prevalence of ED increases with age [21], but the use of ED medication peaks around 65–69 years. This is consistent with previous studies suggesting that among those having ED, the odds of receiving ED treatment decreases with increasing age [5, 18].

The risk score variable was applied in order to focus the analysis on the sociodemographic factors rather than the individual clinical ones, while yet controlling as much as possible for the variation in ED medication use caused by variation in medical needs. The variables included in the risk score were chosen to represent known risk factors for ED such as hypertension, obesity, diabetes mellitus, dyslipidaemia and depression [13, 21, 22, 24] as well as contraindications for using ED medication such as use of vasodilators and previous diagnosis of ischemic heart disease [24, 28]. Most of the associations pointed in the expected direction. One exception was the presence of a diabetes diagnosis at hospital discharge, which was negatively associated with pharmacy dispensing of ED medication in both age groups even though diabetes is a risk factor for ED. Most likely, hospitalization indicates a form of diabetes which is severe enough to signify altered sexual practices. Pharmacy dispensing of oral antidiabetics was, however, positively associated with the use of ED medication, indicating the relevance of diabetes for ED medication use among those less severely ill.

Addition of sociodemographic variables to the regression model increased the discriminatory accuracy in both age groups, indicating that these factors play a role in discriminating between men filling and not filling prescriptions of ED medication. The increase was larger among the elderly men. In both groups, pharmacy dispensing of ED medication was most prevalent among those with high levels of education and income. As this gradient is opposite to that of ED prevalence [13], it implies socioeconomic inequity in the pharmacy dispensing of ED medication. This is consistent with several previous studies [7–12]. The gradient is partly expected since these medications are not reimbursed in Sweden.

In both age groups, pharmacy dispensing of ED medication was more common among divorced and widowed men than among those married. This is contrary to several studies, which have shown higher ED medication use among men who are married or report availability of a sexual partner than among those without sexual partner [8–10]. It should, however, be noted that the marital status variable in the present study does not identify couples that are cohabiting without being married, common in Sweden, and can thus not truly distinguish between those who live with and without a partner. Based on our analysis, it seems unlikely that the associations between pharmacy dispensing of ED medication and socioeconomic position, marital status and country of birth (among the middle-aged men) can entirely be explained by differences in known medical risk factors or contraindications for ED medication use, particularly among the older men where addition of the sociodemographic variables to the regression model added more discriminatory accuracy than among the middle-aged men.

Social scientists have called attention to the increasing role of the market and consumer culture in shaping treatment choices, especially for drugs that can be used to maximize and enhance vitality [29]. According to this view, patients have become active consumers that make choices on the basis of desires that can appear trivial and even irrational. Since repairing and enhancing sexual function by medication use can be regarded as a way to repair and enhance masculinity itself [30] and to secure successful ageing [31], such desires could help explain the increase in prevalence seen in our study. Indeed, ED medication has been marketed as an enhancement product for sexual performance rather than a treatment for a specific medical condition [32]. It is possible that these desires tend to exert different effects in different population groups and that they influence prescribers approach to different patients presenting with ED in different ways. Furthermore, analysis of promotion and discourse surrounding ED medication has shown how the drugs have been marketed using country-specific cultural imaginaries that resonate with middle and upper middle class life, e.g. in Sweden with pictures of scenic landscapes and a particular summer holiday life style in coastal settings [33]. This situation—possibly in combination with the fact that patients’ have to pay for ED medications out of their own pocket—may contribute to explain why the prevalence of pharmacy dispensing of ED medication does not follow the same pattern as ED. The peak prevalence of dispensed prescriptions seen in men who have recently retired and are in the process of redefining their identities away from the labour market, combined with the socioeconomic gradient in our material, calls for further exploration of the possible commercial and cultural driving forces for ED dispensing, in line with the above reasoning.

### Methodological considerations

This study was performed on a large population-based database, and the data contains all prescription fills of ED medication made at Swedish pharmacies by men in Scania during the study period. However, ED medications are known to be subjected to illegal sale over the Internet, and it should be noted that the extent and distribution of this information bias could not be evaluated in this study.

The individual-level analysis covers the year 2006, and the patterns of pharmacy dispensing of ED medication may have changed across the years. However, our analysis is combined with information on national trends for pharmacy dispensing of ED medication. In both age groups, aggregated data show slightly higher prevalence’s than the individual-level data. The main reason for this is that the aggregated data cover all ED medications whereas the individual data only include PDE5I. However, the PDE5I are first-line treatment for ED [28] and represent a large proportion of the sales [5]. Also, the aggregated-level data cover the entire Sweden whereas the individual level data refer only to the region Scania.

The study population was divided into two age strata with the age of 65 years as cut point. This approach was chosen since 65 years is the age for retirement in Sweden and socioeconomic patterns may be different among working and retired men. Altogether, the study population contained the age groups with the highest prevalence of pharmacy dispensing of ED medication. Men aged 80 years or older were not included, mainly because reliable register data concerning educational level are not available for this age group.

The measure of socioeconomic position was created by combining information on educational level and individualised household income. This enabled analysis of the combined effect of being privileged or disadvantaged in respect to these two variables, rather than controlling for one while analysing the other. This variable has some missing data (5.6%) owing to educational level being unknown for some of the participants. Complete case analysis was applied in the regression analyses since the level of missing was relatively low.

The DA of the regression models applied in this analysis is relatively limited. This may be an indication that important variables are missing or that there is heterogeneity in the population that cannot be captured by conventional quantitative epidemiological methods [34].

### Conclusions

The proportion of Swedish men filling prescriptions for ED medication increased between 2006 and 2016. Pharmacy dispensing of ED medication seems linked to the individuals’ socioeconomic position, age and marital status suggesting sociodemographic disparities in the pharmacy dispensing targeting sexual function. However, the low DA of the associations shows the limited capacity of these factors to predict ED medication use at the individual level.
